# Integrated Multi‐Omic Analyses Uncover a Regulatory Link Between Photosynthesis and Drought Tolerance in Field‐Grown Sorghum

**DOI:** 10.1111/pce.70649

**Published:** 2026-06-07

**Authors:** Li'ang Yu, Giovanni Melandri, Anna C. Nelson Dittrich, Hamada AbdElgawad, Gerrit T. S. Beemster, Ciara Garcia, A. Elizabeth Arnold, Brian D. Gregory, Duke Pauli, Andrew D. L. Nelson

**Affiliations:** ^1^ Boyce Thompson Institute Cornell University Ithaca New York USA; ^2^ School of Plant Sciences University of Arizona Tucson Arizona USA; ^3^ Center for Agroecosystem Research in the Desert (ARID) University of Arizona Tucson Arizona USA; ^4^ Integrated Molecular Plant Physiology Research University of Antwerp Antwerp Belgium; ^5^ Department of Biology University of Pennsylvania Philadelphia Pennsylvania USA

**Keywords:** drought response, oxidative stress metabolites, photosynthesis, sorghum, systems biology

## Abstract

Global warming and increasing water scarcity pose major challenges to agriculture, emphasising the need to translate stress‐biology insights into the development of drought‐resilient cultivars. In this study, we evaluated a panel of six diverse sorghum (*Sorghum bicolour*) accessions grown under field‐imposed drought conditions in central Arizona, integrating physiological, transcriptomic and oxidative stress measurements over a 7‐week period. Using network analyses informed by co‐expression correlations, transcription factor (TF) binding motif signatures and protein–protein interactions, we identified a drought‐associated module strongly correlated with photosynthetic capacity. This module contained a stress‐responsive TF, *SbDof8* (referred to here as *SbCDF2/3‐like, or SbCDF2/3L)*, as a highly connected hub gene, and CDF2/3‐associated binding motifs were over‐represented in the promoters of co‐expressed module members. These co‐expressed members were enriched for stress response, metabolic and photosynthesis‐related processes, and consistently maintain higher expression under drought in tolerant compared to sensitive accessions. Analysis of an independent sorghum drought time‐course dataset comprised of two unique accessions revealed concordant expression patterns of *SbCDF2/3L* and photosynthesis‐associated module genes between drought‐tolerant and drought‐sensitive genotypes, reinforcing the robustness of this regulatory module. Together, our results highlight a subset of photosystem I (PSI)–related genes, including light‐harvesting proteins, PSI subunits and importantly, a potential drought‐responsive transcriptional regulator that are collectively upregulated in resilient accessions as a means of coping with drought response. These data highlight promising breeding targets for improving drought resilience and biomass productivity in sorghum under field conditions.

## Summary statement

By integrating a large set of physiological, transcriptomic and oxidative stress data from six diverse *Sorghum bicolour* accessions grown under field drought conditions, this study identified a suite of genes, controlled by an uncharacterised Dof transcription factor, associated with drought resilience.

## 1 Introduction

*Sorghum bicolour* (L.) Moench is the fifth most important cereal crop worldwide and is broadly grown for grain, brewing and cellulosic biomass production (Sanjana Reddy 2017). As a member of the *Poaceae* and a close relative of maize, sorghum is believed to have been domesticated around 4000–6000 years ago in Africa, and to have then diverged into a variety of landraces with diversified morphological variation due to selection across broad geographical locations (Morris et al. 2013). Most sorghum cultivars were historically, and are currently, grown in arid and semi‐arid areas due to their tolerance to limited irrigation during growth. However, when exposed to prolonged and severe (Chen et al. 2025) water deficit, sorghum experiences a reduction in a number of agronomic traits such as biomass and grain yield (Abreha et al. 2021). These traits are believed to be controlled by multiple genes and influenced by a number of developmental and environmental cues (Singh et al. 2025). Thus, a better understanding of the genetic mechanisms allowing sorghum to interpret and coordinate responses to environmental cues is essential for maintaining yield under stress conditions (Alizadeh et al. 2020).

Drought induces numerous unfavourable phenotypic and physiological changes, such as reduced yield, altered plant growth and increased susceptibility to biotic stressors. Plant phenotypic responses to reduced water availability are associated with physiological and molecular responses. Plants with greater drought tolerance typically exhibit increased water use efficiency, which is often achieved through reduced transpiration and consequently leads to lower net CO_2_ assimilation. Environmental stress often disrupts redox homoeostasis and leads to the accumulation of reactive oxygen species (ROS), which in turn leads to an increase in metabolites and oxidative stress enzyme activity levels as a means of ameliorating stress, as commonly seen by the increased levels of specific soluble sugars and amino acids used as osmoprotectants (Hasanuzzaman et al. 2013; Zargar et al. 2017; Fàbregas and Fernie 2019). These phenotypic, physiological and metabolic traits, when combined with additional molecular data, can facilitate the identification of drought tolerance mechanisms linked to enhanced performance.

Drought responses are coordinated at the cellular level through a complex set of regulatory control points, guided by the activation and repression of transcription factors (TFs) that in turn regulate the expression of their target genes (Zhu 2016; J.‐S. Kim et al. 2024). These regulatory networks receive and interpret cues from the environment and govern the transcriptional cascades that link stress signalling to downstream defence and adaptation mechanisms that ultimately determine the effect on phenotypes such as yield (Sato et al. 2024). While large population‐level studies of sorghum have identified strong drought tolerance phenotypes useful for breeding and selection (Ortiz and Salas‐Fernandez 2022; Tsehaye et al. 2024), the crosstalk between many upstream regulatory genes and downstream defence genes and yield‐related traits remains unclear and largely unexplored. Systems‐level approaches have been quite successful at identifying the elements that influence stress and yield in several crop species (Bang et al. 2019; Manna et al. 2021; Yu et al. 2024), although much of this work is often done in a single crop accession.

One family of plant‐specific TFs, the DNA‐binding with one finger (Dof) family, has been observed in a number of species to directly couple environmental signalling with the transcriptional regulation of the photosynthetic machinery. Unlike general stress regulators, Dofs appear to exert specific control over both the light reactions and carbon fixation pathways. For instance, the Maize Dof1 TF has been observed to activate expression of the phosphoenolpyruvate carboxylase genes in both rice and *Arabidopsis* via heterologous expression (Kurai et al. 2011). This induction was more pronounced under limited nitrogen conditions and resulted in increased biomass and carbon assimilation. The rice atypical Dof, OsDes1, was recently shown to directly bind the promoter of a nuclear‐encoded member of the electron transport chain machinery, Rieske FeS (*OsPetC*), activating its expression and thereby conferring a ‘stay‐green’ phenotype fundamental to yield maintenance (Qiu et al. 2024). However, this Dof regulatory capacity is nuanced. In wheat, TaDof7.6 inhibits key C4‐photosynthesis‐associated enzymes (e.g., PPDK); suppression of TaDof7.6 led to enhanced photosynthetic capacity and increased grain filling (P. Zhang et al. 2025). Furthermore, Dofs have been reported to be upregulated during mild drought stress in maize (Nelissen et al. 2018). Finally, a specific subclass of Dofs, the cycling Dofs (CDFs), has also been shown to modulate light‐regulated genes in a circadian fashion (Saibo et al. 2009). Interestingly, while CDFs, such as *Arabidopsis* CDF1 and CDF5 (Henriques et al. 2017; Furihata et al. 2025), have primarily been reported as regulators of flowering time, *Arabidopsis* CDF3 has been observed to enhance photosynthetic capacity under abiotic stress in *Arabidopsis* (Corrales et al. 2017), with ectopic expression resulting in enhanced biomass, yield and carbon/nitrogen assimilation in tomato. The Dof family integrates environmental cues into plant photosynthesis and core metabolism. However, which specific members perform these functions remains unclear.

To identify the genetic basis by which sorghum responds to drought stress, we leveraged a systems biology approach to study six phylogenetically distinct sorghum accessions with different levels of drought tolerance in field conditions (Boatwright et al. 2022). We collected matched transcriptomic, oxidative stress status, photosynthetic and whole plant phenotypic data at multiple points throughout the season to obtain an integrated understanding of the responses to soil water conditions. We uncovered a suite of genes enriched for photosynthesis function in a co‐expression network that was associated with genotypic factors, where the tolerant and sensitive accessions performed differently. Based on co‐expression and promoter binding element enrichment, we hypothesised that a homologue of the CDF (SbCDF2/3L) subfamily acts as a potential key regulator linking photosynthesis and stress response. To test the hypothesis, we examined the profile of SbCDF2/3L putative target genes, which exhibited a consistent drought‐response pattern across both our field experiment and an independent sorghum drought time‐course dataset (Varoquaux et al. 2019). Drought‐tolerant genotypes maintained elevated expression under water stress relative to control conditions of both *SbCDF2/3L* and a functionally coupled group of photosynthetic genes coordinating carbon fixation, photosystem I assembly and electron transport. Elevated expression of these genes in drought‐tolerant accessions was associated with higher photosynthetic capacity, whereas sensitive genotypes showed pronounced downregulation along with strongly reduced photosynthetic performance. Overall, these data support this cohort of genes as strong candidates for targeted breeding aimed at sustaining biomass accumulation and productivity under drought conditions.

## 2 Methods and Materials

### 2.1 Sorghum Growth and Field Experiment Setup

Six selected sorghum accessions (PI 533871: M1, PI 533961: DL/59/1530, PI 656041: 80M, PI 656053: N290‐B, PI 656076: SC1271 and PI 656096: SC391) were grown at the University of Arizona Maricopa Agricultural Center in Maricopa, AZ, US (33°04’37”N, 111°58’26”W). Measurements of soil volume metric water content (SVWC) were collected on a 2–3 day basis at four depths (30, 50, 70 and 90 cm) using a field‐calibrated neutron moisture probe (Model 503, CPN, Martinez, CA, U.S.) along with meteorological data that were collected by an automated Arizona Meteorological Network (AZMET) weather station. All plants were well‐watered (WW) at the same rate (~24%, SVWC, corresponding approximately to the soil field capacity) until flag leaf appearance in the whorl (~47 days after planting). After this point in time, two replicates of the six genotypes were maintained at WW conditions for the remainder of the season, while three replicates were subjected to water deficit stress (water‐limited [WL]) to mimic drought; this corresponded to an SVWC of 16%. Destructive plant phenotyping was conducted at harvest, beginning on 4 November 2020, when the plants had reached full maturity. Measured traits included above‐ground biomass, root biomass, the ratio between the two and root endophytic mycobiome. For other leaf tissues used for time‐series molecular phenotypes that include RNA‐seq and oxidative stress‐related biochemical quantification, samples were collected weekly (on Thursdays) for 7 weeks (week 1‐week 7, from 13 August to 24 September 2020).

### 2.2 Sorghum Biomass and Morphological Traits Measurement

Five plants of each accession under two conditions were sampled during early November 2020 from each plot. Aboveground biomass was collected by cutting plants approximately 5 cm above the soil surface and included stems, leaves and panicles. Harvested aboveground tissues were placed in bags and oven‐dried at 60°C for 2 days before weight measurement. Root systems from the same plants were excavated, gently cleaned to remove adhering soil, and oven‐dried at 60°C for 2 days. Total plant biomass and root‐to‐shoot ratio were calculated as the sum of aboveground and root dry weight (DW), and ratio of root DW divided by shoot DW. For root morphological traits, root length was defined as the distance from the cut stem base to the tip of the longest root, whereas maximum root width was defined as the greatest lateral distance between the two outermost root tips.

### 2.3 Sorghum Microbiome Data Analysis

Sorghum root endophytic (ENDO) mycobiome data were derived from publicly available analyses on these same field experiments (Garcia et al. 2024). Briefly, six focal accessions with three biological replicates were used to characterise root‐associated endophytic bacteria and fungi under WW and WL conditions at the terminal stage of the same 2020 field trial. Following DNA extraction, read mapping and taxonomic quantification (Garcia et al. 2024), a total of 515 bacterial and 46 fungal operational taxonomic units (OTUs) were identified. In this study, OTUs abundance profiles for each genotype under both watering conditions were used as input for principal component analysis (PCA) in R (Core Team R. R 2013).

### 2.4 Sorghum Photosynthesis‐Related Traits Measurement

Photosynthesis‐related traits were measured using a LI‐6800 portable photosynthesis system (LI‐COR Biosciences, Lincoln, NE, USA) with five replicates per accession over four time points under two watering conditions. Measurements were conducted in the morning (09:00–10:00) and afternoon (13:00–14:00) under the following chamber conditions: air flow rate of 600 µmol s^−1^, mixing fan speed of 12 000 rpm, reference CO_2_ concentration of 420 ppm and photosynthetically active radiation (PAR) light intensity was set at 2000 µmol m^−2^ s^−1^ photosynthetic photon flux density. The six youngest fully expanded leaves on the main stem of each plant were selected for the measurements, and data were logged as soon as assimilation and stomatal conductance curves (monitored in real time on the instrument display) reached a plateau (on average, 30 s after a leaf was clamped onto). Chlorophyll fluorescence was measured using a multiphase flash fluorometer head. Light‐adapted fluorescence parameters (Fs, Fm’, Fv’) were measured using the multi‐phase flash protocol and a saturating pulse at ~10 300 µmol m^−2^ s^−1^.

### 2.5 Sorghum Oxidative Stress‐Related Biochemical Measurements

Leaf‐level oxidative stress marker and molecular antioxidant content, as well as antioxidant enzyme and photorespiration enzyme activity (replicate = 5, with six leaf discs per sample), were analysed following established protocols in a previous study (Melandri et al. 2020). Oxidative stress markers, such as malondialdehyde (MDA) and protein carbonyl content, were measured to assess lipid peroxidation and protein oxidation (Hodges et al. 1999). Photorespiration‐related enzyme activities, glycolate oxidase (GOX) and hydroxypyruvate reductase (HPR), were assessed based on established methodologies (Schwitzguebel and Siegenthaler 1984). Lipoxygenase (LOX) was extracted in potassium phosphate buffer (pH 7.0), and its activity was measured by changes in conjugated dienes (Steczko et al. 1991). Total antioxidant capacity (TAC), total polyphenol content (Poly) and flavonoids were evaluated using standard approaches (Benzie and Strain 1999). Enzyme activities of key antioxidants, including ascorbate peroxidase (APX), dehydroascorbate reductase (DHAR), monodehydroascorbate reductase, glutathione reductase, peroxidase (POX), superoxide dismutase (SOD), glutathione peroxidase (GPX), catalase (CAT), ascorbate oxidase (AO) and glutathione *S*‐transferase (GST), were determined using microplate‐based kinetic assays (Dhindsa et al. 1982).

### 2.6 RNA Extraction and Library Construction

Approximately five flash‐frozen sorghum ¼‐inch leaf discs, derived from five individual plants within the same plot, were used as starting material for RNA extractions. Replication occurred at the plot level to better account for field and treatment effects. RNA extraction was performed using the RNeasy Plant Mini kit (Qiagen #74904) and RNase‐free DNase set (Qiagen #79254) according to the manufacturer's instructions. Depending on the total RNA yield for each sample, 2.5–10 µg total RNA was used as starting material for mRNA enrichment using the Dynabeads mRNA purification kit (Invitrogen #61006) according to the manufacturer's instructions, except with the addition of a second binding and elution step to further enrich mRNA. RNA‐seq libraries were then constructed from 10 to 60 ng mRNA per sample using the Amaryllis Nucleics YourSeq Duet Full Transcript library prep kit with combinatorial dual indexes (now Active Motif #23001 or #23002) according to the manufacturer's instructions. Prepared libraries were sequenced by Novogene with Illumina NextSeq. 500 (PE × 150 bp).

### 2.7 Transcriptome Data Processing

Sorghum RNA‐reads of six accessions were aligned to the sorghum reference genome (McCormick et al. 2018) using Hisat2 with default settings (D. Kim et al. 2019). FeatureCounts was used for quantification using the gene ID as the meta‐level to count reads (v2.0.1, parameter: ‐s 1 ‐p ‐t mRNA ‐g ID ‐O). Read counts of 34 130 primary transcripts were normalised using DESeq. 2 after the removal of transcripts with less than an average of one count across 96 samples (Liao et al. 2013). We incorporated the time point (week 1, week 3, week 5 and week 7), accession IDs (A: M1, B: DL/59/1530, C: 80M, D: N290‐B, E: SC1271 and F: SC391), and watering conditions (control and drought) as the experimental design factors. Condition‐wise differentially expressed genes (DEGs) over each time point per accession were identified using the cutoff of F*DR* < 0.5 (Love et al. 2014). A log_2_‐transformed fold change (FC) greater than 1 was used to filter up‐regulated genes, and a less than −1 log_2_FC filtered the down‐regulated genes. RNA reads of a published large‐scale sorghum drought transcriptome dataset covering 8‐week post‐flowering drought experiment on two cultivars (Varoquaux et al. 2019, GEO: http://www.ncbi.nlm.nih.gov/geo/) under accession no. GSE128441 was processed using the same pipeline above. For both public data and RNA‐seq in the current experiment, transcripts per million (TPM) score was calculated for read counts normalisation for comparing relative expression of genes among samples in each experiment.

### 2.8 Construction of Co‐Expression Networks

The co‐expression network was constructed using the WGCNA R package with variance‐stabilising transformed normalised read counts from 34 130 transcripts across 96 samples (Langfelder and Horvath 2008; Love et al. 2014). Initially, a soft threshold (Beta score) was applied to fit the scale‐free topology model assumption, which generates a minimum number to make the index curve flatten out, reaching a high value (> 0.85, Yu et al. 2024). Further, the adjacency matrix represented by gene expression was transformed into a topological overlap matrix (TOM) to classify genes using a dynamic tree‐cut approach. Finally, genes that shared a high‐confidence correlation (abs(coefficient) > 0.8) were merged into the same co‐expression network module, representing clusters of genes with similar expression profiles. (parameter: deepsplit = 4, tree cut height = 0.2.) The pairwise Pearson correlation (PC) between each gene from modules and the module‐wise eigengene values was tested to characterise each gene's module membership (MM) under the same module using the signedKME function of WGCNA. The weight score, a parameter to denote the robustness of connections of each edge, was filtered by taking genes associated with the top 10% of high‐confidence weight ranking connections for downstream functional analysis. Lastly, the metabolite profiles, oxidative stress‐related enzyme activities and LI‐6800‐derived traits were integrated into the network using PC between gene expression and these molecular phenotypes. Trait‐correlated modules were selected based on their correlation level between MM scores and phenotypes (cutoff: *r* > 0.5, or *r* < −0.5, *p* value < 0.05). The correlation between gene expression and phenotypes was estimated as gene significance (GS) to interpret the importance of genes related to each molecular phenotype.

### 2.9 Functional and Regulatory Network Analyses

To study TFs and their downstream target genes (protein‐DNA interactions), TFs and protein kinases from each co‐expression module were predicted using the iTAK tool (http://itak.feilab.net/cgi-bin/itak/index.cgi; Zheng et al. 2016) based on conserved protein‐coding domains (Zheng et al. 2016). To identify potential upstream regulators of genes in each module, we performed a motif enrichment analysis for the 2 kb upstream promoter regions of genes in the same module using AME (‐‐scoring avg, ‐‐method fisher and ‐‐hit‐lo‐fraction 0.25) and the Arabidopsis DAPseq consensus motif database (Bailey et al. 2015; O'Malley et al. 2016). The protein‐protein interactions (PPIs) among genes present in the co‐expression network were acquired from the STRING database (https://string-db.org/, Szklarczyk et al. 2023) as the third layer of evidence to infer the functions or pathways of modules (parameter: confidence level: > 0.7, source: experiments and database source (Mering 2003). As a result, co‐expression patterns, PPIs and TF‐binding interactions were merged into an integrated network using the ‘union’ function within Cytoscape (https://cytoscape.org/; Shannon et al. 2003).

### 2.10 Gene Ontology (GO) and Pathway Enrichment Analyses

Functional enrichment analysis of pathways was performed using Kyoto Encyclopaedia of Genes and Genomes database (KEGG: https://www.genome.jp/kegg/; Kanehisa et al. 2016) and GO to infer the biological function of genes associated with those assigned to co‐expression modules or found to be differentially abundant between control and drought conditions. ShinyGO (https://bioinformatics.sdstate.edu/go77) was used to perform GO and KEGG enrichment analyses based on the sorghum gene functional annotations provided within the website (Ge et al. 2019).

### 2.11 Phylogenetic Assessment of SbCDF2/3‐Like Genes

Sorghum Dof homologues were identified by reciprocal best BLAST using CoGe (genomevolution. org; Nelson et al. 2018) with *Arabidopsis* CDFs 1, 2 and 3 as query (AT5G62430, AT5G39660 and AT3G47500, respectively) with an E‐value of 1e−10. *S. bicolour* v3.1 (Phytozome, McCormick et al. 2018), *Oryza sativa japonica* (Ensembl Release 36; Kawahara et al. 2013) and TAIR10 genomes and annotations were used for searching for homologous sequences. Rice and Arabidopsis Dofs were named according to nomenclature in the previous study (Lijavetzky et al. 2003), with Sorghum labelling derived from Kushwaha et al. (2011). To infer relationships, multiple sequence alignments were generated using the Geneious multiple sequence alignment algorithm within Geneious v2023.0 (www.geneious.com) with default settings on both the core Dof domain (plus 10AA on either side) and full‐length protein sequences. RAxML (Stamatakis 2014) was used to infer phylogenies (GTR‐Gamma with rapid bootstrapping and 100 bootstraps). A larger Dof subfamily, comprised of both D_1_ + d_1_ and D_2_ + d_2_ subfamilies, was used to place sorghum Dofs within the CDF‐like family, with a secondary analysis on D_1_ + d_1_‐specific genes (which contain the *Arabidopsis* CDF‐specific genes).

### 2.12 Data Visualisation and Usage of Databases

All statistical visualisations were generated in R (v4.3.2) with multiple packages (Core Team R, R 2013). Briefly, plots include the line, boxplot, PCA and enrichment graphs generated by the ‘ggplot2’ package, and heatmaps using the ‘pheatmap’ package. R Scripts used for plots were deposited in https://rpubs.com/LeonYu/Sorghum-comparative-transcriptomics. The integrated gene networks containing TF‐binding, PPIs and co‐expression derived from WGCNA were generated using Cytoscape.

## 3 Results

### 3.1 Diverse Phenotypic Response to Drought Among Sorghum Accessions

**Figure 1:**
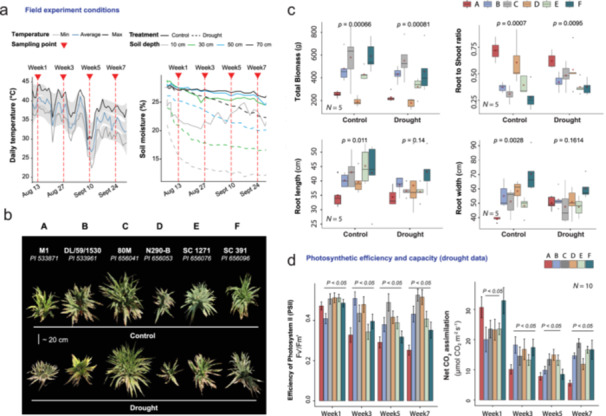
Drought response diversity of selected sorghum genotypes. (a) Daily temperatures (left) and soil moisture at different soil depths (soil volume metric water content, %; right) during a field experiment conducted in Maricopa, AZ, during the summer of 2020. (b) Morphological responses of sorghum accessions under control and drought conditions, showing plant architecture at the terminal field sampling stage. (c) Physiological measurements of whole‐plant biomass (top left), root‐shoot‐ratio (RSR; top right) and root morphological traits (bottom) under each condition (*N* = 5 per accession) were compared using a Kruskal test. (d) Bar plots showing photosynthetic efficiency and capacity under drought conditions, measured as Fv′/Fm′ and net CO_2_ assimilation, respectively, at each time point for drought‐treated plants (*N* = 10 per accession). Pairwise Wilcoxon tests were used to compare each accession relative to ‘A’ (*p* < 0.05).

All six sorghum accessions were selected from the sorghum diversity panel (Boatwright et al. 2022) based on seed viability, genetic distinctiveness and phenotypic variation to drought treatment (shoot and root biomass, plant height and yield). Among the selected lines, four accessions (A, B, E and F) were African accessions, while the remainder (C and D) were U.S. breeding accessions. Arizona summer field conditions are well known for high temperatures and low humidity. Indeed, daytime temperatures peaked near 45°C in the first week of collections, with lows around 30°C observed briefly in the third week (Figure 1a, left). To assess plant performance of these accessions under drought conditions, plants were grown in replicated plots as part of a large field trial. All plants were irrigated at normal rates (control conditions) until the reproductive stage (appearance of the flag leaf), after which a drought treatment was implemented by reducing irrigation to a subset of plots, as determined by continuous monitoring of soil water content (Figure 1a, right). Phenotypic measurements and molecular sampling (transcriptomics and oxidative stress biochemical traits) occurred during the 1st, 3rd, 5th, and 7th week after the beginning of the drought treatment while the stage had not transitioned into grain filling (Figure 1a), with destructive measurements (e.g., biomass and root microbiome composition) at the beginning of November 2020 during the harvest season.

Initially, overall plant‐level phenotypes confirmed that the reduction in irrigation was sufficient to observe drought stress responses in the panel. In particular, treatment effects were readily apparent and pronounced at the end of the season (16 September 2020), with the M1 (‘A’) accession showing the most pronounced leaf browning (Figure 1b). Given that root‐associated microbiome responses are closely linked to plant drought responses, we used microbiome composition as a complementary indicator of drought treatment effects. Endophytic root microbiome bacterial and fungal composition from a public dataset developed from this panel (Garcia et al. 2024) was analysed using a dimensional reduction approach. These data suggest that the microbiome distribution among samples was impacted by water treatment, with clear separation between drought‐ and non‐treated samples (Figure S1a). We found that the ‘A’ accession exhibited fewer degrees of separation between control and drought conditions than other accessions (Figure S1a).

We further used biomass‐related measurements and two root morphological measurements to estimate the level of drought tolerance at the terminal stage of the experiment (Figure 1c). When comparing drought and control data separately, we observed significant variation among accessions over four traits (*p* < 0.05). In particular, the ‘A’ (M1) and ‘D’ (N290‐B) accessions had the lowest total biomass under both control and drought conditions (*p* < 0.05), potentially indicating either poor adaptation to both heat and drought stress or inherently lower biomass associated with these genetic backgrounds (Figure 1c, top left). As another gross physiological indicator, we calculated the root‐shoot‐ratio (RSR) that reflects biomass allocation strategies during stress and growth. We observed significant variation under both conditions among accessions (*p* < 0.05), in particular with the highest RSR in accession ‘A’ under both conditions (Figure 1C, top right). When comparing RSR between drought and control, accession ‘A’ showed the greatest reduction under drought, in contrast to accessions ‘C’ and ‘F’, which displayed an increase in RSR under drought (Figure S1b), suggesting a reduced ability to acquire resources in ‘A’ during drought stress. For root morphological phenotypes, ‘A’ exhibited significantly lower length and width compared with the other five accessions under control conditions (Figure 1d, *p* < 0.05), but these differences became less pronounced under drought stress (Figure 1d). When drought tolerance was evaluated using drought/control biomass ratios, ‘A’ (M1) and ‘F’ (SC 391) displayed a pronounced reduction of two distinct indicators, total biomass and leaf weight (Figure S1a), whereas ‘A’ showed an increase in root‐related morphological features.

Lastly, we examined photosynthetic efficiency and capacity to develop a clearer understanding of the leaf‐level responses to treatment (Figure 1d and Figure S1c). Here, photosynthetic efficiency and capacity were estimated by calculating maximum photosystem II efficiency in light conditions (Fv’/Fm’) and net CO_2_ assimilation (A_net_; Figure 1d). These data indicate that the ‘A’ accession displayed hallmarks of drought stress and heat stress, particularly at the later time points (week 5 and week 7, Figure 1d and Figure S1c), whereas other accessions, such as ‘B’ and ‘C’, showed a more robust response to drought conditions. These combined morphological and physiological data suggest that these six accessions display phenotypic diversity to drought stress, with B and C being more tolerant and A and F being more sensitive. Thus, these phenotypic classifications should allow for the uncovering of molecular mechanisms by which sorghum responds to water‐limiting conditions.

### 3.2 Sorghum Accessions Displayed Molecular Response Variation During Drought Stress

**Figure 2:**
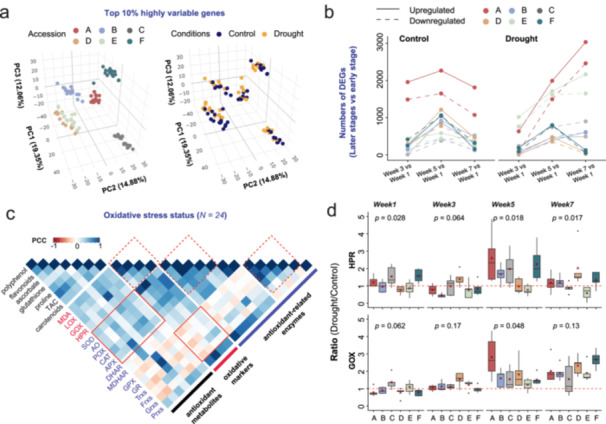
Molecular response to drought among sorghum accessions. (a) Principal component analysis (PCA) of the top 10% variable transcripts (3413) using DESeq. 2 normalised reads with comparison among accessions and two watering conditions. (b) A line graph representing the number of up‐regulated (solid line) or down‐regulated (dashed line) genes under the two watering conditions. (c) Pairwise Pearson correlation coefficient (PCC) of 24 chemicals associated with antioxidant metabolites, antioxidant enzymes and oxidative stress markers was displayed by a heatmap with the three chemical classes denoted. Red dashed boxes show the correlation of metabolites belonging to the same class, and red solid line boxes highlight the correlation between two classes. (d) Representative two oxidative markers (HPR and GOX) from (d) were selected to display the ratio between drought/control across time for each of the six genotypes. [Color figure can be viewed at wileyonlinelibrary.com]

To characterise the molecular responses of drought stress in our panel, we next assessed the change in transcript abundance over the 2 months of the treatment (week 1, 3, 5 and 7) using RNA‐seq. Initial quality control revealed a high correlation between replicates (PC coefficient; *r* > 0.95). Using hierarchical clustering and PC analysis of global transcript abundance (*N* = 22 314, mean TPM > 1), the 96 samples clustered into subgroups that fit their genotypic classification (Figure S2a). Consistent with a PCA based on the top 10% most variable genes, we observed clear separation among accessions, as well as between drought and control samples for some accessions. (Figure 2a), In all, these data point to genotype being the largest driver of separation, followed by treatment and time points (Figure S2b), for most accessions.

To characterise transcriptomic responses, we first performed time‐series differentially expressed gene analyses (*FDR* < 0.05) comparing later stages (weeks 3, 5 and 7) to week 1 within each accession under both conditions to minimise potentially confounding heat effects in the control samples. Interestingly, reductions in photosynthetic efficiency and biomass under drought stress (Figure 1c,d) did not necessarily correspond to upregulation of stress‐response pathways. When comparing ‘A’, an accession that showed reduction in both biomass and photosynthetic efficiency, to ‘C’, which was robust to drought by both measurements, we observed remarkably different enrichment molecular response terms (Table S1). Accessions ‘D’ and ‘F’ showed the fewest differentially expressed genes (DEGs) under prolonged stress, whereas ‘A’ and ‘E’ exhibited the most (Figure 2b). Accessions ‘B’, ‘C’ and ‘D’ showed minimal enrichment of upregulated genes with stress response GO terms, in strong contrast to accessions ‘A’, ‘E’, and, to a lesser extent, ‘F’ (Table S1). Interestingly, accession ‘E’ showed downregulation of a number of abiotic stress response terms when comparing week 7 to week 1 that was not seen under control conditions. Highlighting the unique responses to treatment across these accessions, minimal overlap was observed for either up‐ or down‐regulated DEGs among the six accessions, consistent with a genotype, rather than a treatment‐driven global transcriptomic response (Figure S2a,c).

To gather additional clues as to how these accessions responded to drought treatment at the molecular level, we next examined the stress‐induced changes in components of oxidative stress response or resolution pathways. Samples for oxidative stress‐related metabolite and transcriptome measurements were collected simultaneously to enable stronger associations. This metabolite profiling dataset comprised oxidative stress markers, antioxidant metabolites and antioxidant‐related enzymes (*N* = 24). Pairwise correlation analysis revealed strong within‐class correlations (Figure 2c), with oxidative stress markers (MDA, LOX, GOX and HPR) showing partial negative correlations with several antioxidant‐related enzymes (e.g., GPX, Trxs and Grxs). In contrast, most antioxidant metabolites were positively correlated with a subset of antioxidant enzymes, including SOD, AO, POX, CAT and APX. Together, correlation in metabolites and related enzymes reveals the expected coordinated oxidative stress response across accessions.

We then compared oxidative stress‐induced response levels, quantified as drought/control ratios, across several representative metabolites. Among oxidative stress markers, we focused on two enzymes, HPR and GOX, which play key roles in the photorespiratory pathway and link photosynthetic carbon metabolism with ROS homoeostasis (Figure 2d; Keech et al. 2017). In addition, four antioxidant components (glutathione, ascorbate, Frxs and GPX) were included, as they protect photosynthetic and photorespiratory processes by maintaining redox balance and scavenging ROS (Figure S3; Sakhno et al. 2019; González et al. 2021). At weeks 5 and 7, we observed pronounced stress responses, with accessions ‘A’ and ‘F’ showing higher accumulation of two oxidative stress markers (Figure 2d) and generally reduced levels of antioxidant enzymes and metabolites compared with the other four accessions (Figure S3). These patterns indicate elevated oxidative stress in accessions ‘A’ and ‘F’, consistent with the sustained upregulation of stress‐responsive DEGs at later stages relative to the initial stage. (Figure 2b and Table S1). Overall, transcriptomic and oxidative stress profiles align with photosynthetic traits and other physiological parameters, highlighting that ‘A’ and ‘F’ are drought‐sensitive, with accession ‘A’ showing the poorest performance across multiple criteria.

### 3.3 Identification of a Drought‐Associated Cohort of Co‐Expressed Transcripts

After characterising molecular and physiological traits and assessing drought sensitivity across accessions, we applied a comparative systems‐level approach to link these traits to underlying molecular pathways. We constructed a co‐expression network using the top 10% most variable transcripts (median absolute deviation) and optimised network parameters by iteratively increasing transcript inclusion to maximise scale‐free topology and minimise unassigned genes (Yu et al. 2024). The final network comprised 22 314 transcripts (65.5% of annotated sorghum genes) grouped into 44 modules, with a low proportion of unassigned transcripts (*N* = 2646) and strong scale‐free topology (*r* = 0.91, slope = −1.93; Table S2).

**Figure 3:**
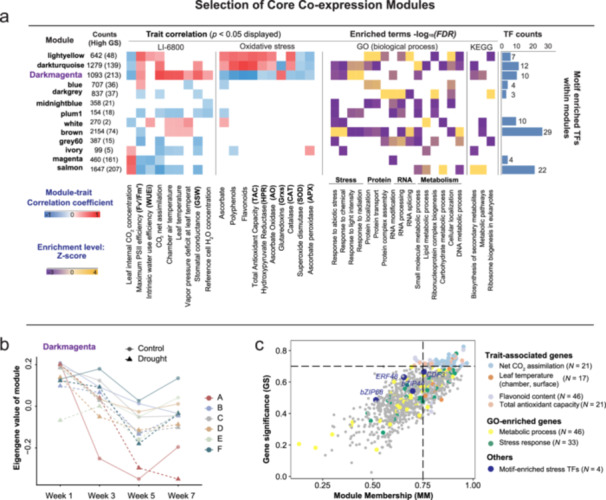
Selection of co‐expression modules contributing to drought tolerance. (a) The number of transcripts per module, correlation between traits and the module (blue and red scale), levels of KEGG and GO enrichment (purple and yellow scale), and the number of TFs with enriched motifs for each module are displayed. Scales for module‐trait correlation values, as well as GO‐enrichment levels, are shown in the bottom left. Only those module‐trait correlations with a *p* value < 0.05 are shown. (b) The eigenvalue of each ‘Darkmagenta’ module, divided by accession, treatment and time, was plotted. (c) The scatter plot shows module membership (MM) and gene significance (GS) of genes in the ‘Darkmagenta’ module, with genes coloured based on their strong expression‐trait correlation, GO enrichment, or annotation as a stress‐responsive transcription factor. [Color figure can be viewed at wileyonlinelibrary.com]

Using this framework, we incorporated phenotypic data into modules using trait‐module correlations. Briefly, the first principal component value (eigenvalue) per module was calculated using the transcript abundance PCA. Leaf‐level physiological measurements and biochemical traits related to oxidative stress status were then correlated with the eigenvalues of each module (Tables S3 and S4), leading to 13 modules with significant trait‐module correlations (*p* < 0.05, Figure 3a, left panel). Several modules (e.g. ‘Lightyellow’, ‘Darkturquiose’ and ‘Darkmagenta’) were strongly correlated with both physiological and biochemical traits. To further prioritise modules, we examined pairwise correlation values between each gene within a module and the associated traits (referred to as GS) to uncover modules harbouring genes with the strongest correlation to traits of interest. This approach identified a suite of genes with high GS (| GS | > 0.65, denoted in Figure 3a; *N* = 1167). In particular, we observed the largest number of genes with strong positive correlation (*N* = 213; Darkmagenta) with photosynthetic parameters and oxidative stress markers. In addition, ‘Darkmagenta’ had the highest proportion of genes that passed the GS threshold relative to the overall module, with the exception of the ‘magenta’ module, which was correlated with a small number of traits. Thus, the ‘Darkmagenta’ module was prioritised for identifying potential drivers of drought stress in this panel.

To gain insights into the biological processes associated with each of these modules, GO and KEGG pathway enrichment analyses were performed (modules with *FDR* < 0.05 are displayed, Figure 3a, middle panel). This approach revealed modules with stress response, translation and metabolic pathways terms. In addition, we performed a motif enrichment analysis using the *Arabidopsis* DAP‐seq motif database (O'Malley et al. 2016) to identify binding motifs for orthologous TFs over‐represented in the promoters of genes in the 13 modules (*FDR* < 0.05). We then determined which of the enriched motifs matched TFs found within the corresponding module, suggesting those TFs may target co‐expressed genes and potentially serve as a hub TF (Figure 3a, right panel). Using the Analysis of Motif Enrichment (AME, Bailey et al. 2015), we found the binding motifs of 10 TFs that were significantly enriched in the 2 kb upstream promoter regions of genes in the ‘Darkmagenta’ module. Of these TFs, 4 are annotated as regulators of abiotic stress (e.g., *bZIP*, *ERF* and *CDF* family TFs; Table S5), agreeing with our inference that this represents a module of stress‐associated genes.

We further examined the ‘Darkmagenta’ module to assess its relationship with genotype, condition and time. Module eigengene profiles revealed a strong genotypic effect, with accession ‘A’ clearly separating from others, particularly at weeks 3–7 (Figure 3b). This pattern is consistent with its distinct leaf‐level physiological responses, suggesting that genes in the ‘Darkmagenta’ module are associated with reduced photosynthetic capacity, increased oxidative stress and drought sensitivity in this genotype (Figures 1, 2). Finally, we examined MM of the transcripts in this module relative to their GS to evaluate whether genes strongly associated with physiological traits also occupy central positions within the module (Figure 3c). We observed a positive correlation between MM and GS for this module, with genes that were correlated with photosynthesis traits (GS > 0.7) also displaying high MM (> 0.75), indicating that trait‐relevant genes tend to be highly connected within the module. In addition, genes annotated with stress response–related GO terms also exhibited elevated MM values (green dots), supporting the functional relevance of this module. Among the four AME‐enriched stress‐response TFs, Sb*CDF2/3L* showed the highest MM and GS, suggesting a prominent role as a regulatory hub linking transcriptional and photosynthetic trait variation (Figure 3c). Collectively, these integrated network analyses identify a core set of highly connected genes with strong trait associations and highlight *CDF2/3L* as a likely hub regulator coordinating stress‐responsive photosynthetic capacity.

### 3.4 *SbCDF2/3L* Binding Motif Associated Genes Exhibit a Drought Stress Response Pattern

**Figure 4:**
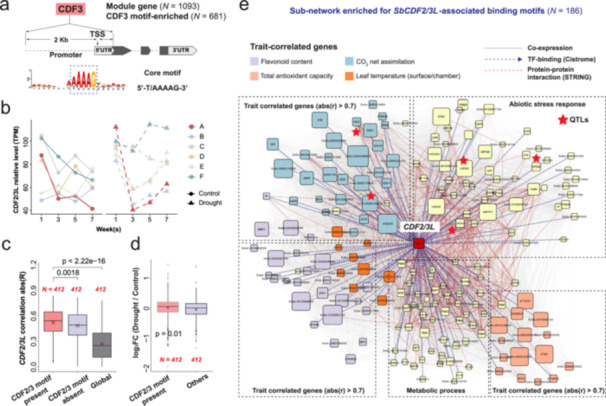
Examination of the photosynthesis and stress response‐related network. (a) Proximal (2 kb upstream) promoter elements for the 1,093 genes in the ‘Darkmagenta’ module were examined for motif enrichment by Analysis of Motif Enrichment (AME). The consensus motif for CDF2/3L in these promoters (bottom left), and the number of CDF2/3L targets within the ‘Darkmagenta’ module, are shown. The core motif for the *Arabidopsis* CDF2/3L is shown to the right. (b) SbCDF2/3L relative abundance (TPM) among genotypes, time points and treatments. (c) The correlation between SbCDF2/3L and predicted (in‐module) target transcripts, non‐SbCDF2/3L target in‐module transcripts, and randomly selected transcripts from outside the module (*N* = 412 for each set) was compared. The number in each group was subset to match the maximum number of non‐SbCDF2/3L predicted targets within the ‘Darkmagenta’ module. Significance between groups is shown (Wilcox test, *p* < 0.05). (d) The log_2_FC(Drought/Control) value of all samples for the CDF2/3L‐associated genes and others from (c) is shown. Significance (shown) was calculated with a Wilcox test. (e) A child network of 230 genes with high module membership was visualised using Cytoscape. Solid grey lines indicate co‐expression only, dashed red lines indicate protein‐protein interactions derived from the STRING database, and the blue dashed and arrowed lines highlight DAP‐seq‐derived putative targets of SbCDF2/3L. Nodes (genes) are coloured based on trait‐association and grouped based on GO‐term enrichment. Red stars indicate genes overlapping net CO_2_ assimilation QTLs from previous GWAS (Ortiz et al. 2017). [Color figure can be viewed at wileyonlinelibrary.com]

Based on the framework described above for identifying core genes within the ‘Darkmagenta’ module, we further examined the hub CDF2/3L TF and its relationship with other trait‐associated genes. This gene was previously annotated as *SbDof8* (Kushwaha et al. 2011), however, phylogenetic analysis and motif similarity based on *Arabidopsis* and rice nomenclature place it in the ‘Cycling Dof’ subfamily (Lijavetzky et al. 2003), sister to *Arabidopsis* CDF2 (AT5G39660) and CDF3 (AT3G47500; Figure S4). Interestingly, this SbCDF2/3L homologue is the only sorghum CDF/Dof in the ‘darkmagenta’ module (Figure S5). The *Arabidopsis* CDF3 (*Arabidopsis* DAP‐seq database ID: C2C2dof_tnt. CDF3, TAIR ID: AT3G47500, sorghum ID: Sobic.002G421900, https://phytozome-next.jgi.doe.gov/) has previously been reported in both *Arabidopsis* and, via heterologous experiments in tomato, as a transcriptional activator that enhances drought and oxidative stress tolerance, carbon and nitrogen metabolism (Noguero et al. 2013; Corrales et al. 2014, 2017; Renau‐Morata et al. 2024). Out of the 1093 genes within the ‘Darkmagenta’ module, 681 (62% in total, e‐value < 0.005) were predicted to be bound by the putative SbCDF2/3L TF in their proximal promoters (Figure 4a). Careful examination of the binding motifs of those 681 putative CDF2/3L‐bound and co‐expressed genes revealed that the consensus motif contains the core CDF2/CDF3 binding motif (5′‐T/AAAAG‐3′; Figure 4a) reported in previous molecular and biochemical studies from maize (Yanagisawa and Schmidt 1999) and in the *Arabidopsis* cistrome database (O'Malley et al. 2016). Interestingly, an examination of Sb*CDF2/3L*'s expression pattern across the 96 sorghum samples revealed a dramatic decrease in the ‘A’ accession in weeks 3–7 under both control and drought conditions (Figure 4b). In addition, we examined the correlation between Sb*CDF2/3L* and each of its predicted targets (*N* = 412, selected by random extraction) in the ‘Darkmagenta’ module, a similar number of non‐CDF2/3L targeted transcripts within the module, as well as randomly selected transcripts from outside of the ‘Darkmagenta’ module (*N* = 412). A significantly higher correlation was observed between *CDF2/3L* and within‐module targets compared with the other two groups (*p* = 0.0018 and *p* < 2.2e−16, Figure 4c). Lastly, we compared the log_2_FC score (Control/Drought) of CDF2/3L putative targets and non‐CDF2/3L targets across all accessions and time points. This comparison revealed a significantly higher log_2_FC (*p* = 0.01) of *CDF3* putative targets (Figure 4d), suggesting these putative target genes are induced under drought conditions in a SbCDF2/3L‐dependent manner.

Motif enrichment and trait associations of genes within the ‘Darkmagenta’ module suggest that SbCDF2/3L functions as a potential central regulator linking drought responses to photosynthetic traits. To further resolve this relationship, we focused on highly trait‐associated transcripts (GS > 0.70; *N* = 82) and those that corresponded to the GO terms enriched within this module (abiotic stress and metabolic process, *N* = 112; Figure 3a). We then integrated TF‐binding and protein–protein interaction (PPI) data on this set of genes to develop a sub‐network of 186 unique genes out of the original 1093 (Figure 4e; Table S6). Of these, 129 were predicted SbCDF2/3L targets (AME). Within this subnetwork, genes were associated with net CO_2_ assimilation (*N* = 27), flavonoid content (*N* = 25), leaf temperature (*N* = 9) and TAC (*N* = 15). GO enrichment further highlighted abiotic stress and metabolic processes, and several genes overlapped with previously reported QTLs for CO_2_ assimilation (Ortiz et al. 2017, Table S6 in the ‘QTL’ column). Furthermore, within this module we observed a large number of PPIs, spanning abiotic stress response, metabolic processes and trait‐correlated genes (Figure 4e). For example, we observed proteins with numerous reported physical interactions between each other and other proteins within the photosynthetic pathway (Mehari et al. 2021; Cheng et al. 2025; Huang et al. 2025; Mering 2003). We also observed PPIs for metabolic regulators that link photorespiration, carbon metabolism and ABA signalling to sustain photosynthesis and growth under low CO_2_ (e.g., PPC2, You et al. 2020), as well as downstream signalling proteins that facilitate appropriate responses under stress (e.g., STN7 and PGR5 [Guangxin et al. 2025; Lee et al. 2025]). Together, these results support SbCDF2/3L as a potential regulator of drought‐responsive gene networks underlying physiological variation in sorghum.

### 3.5 Validating the SbCDF2/3L Regulatory Response in Publicly Available Field Stress Data

**Figure 5:**
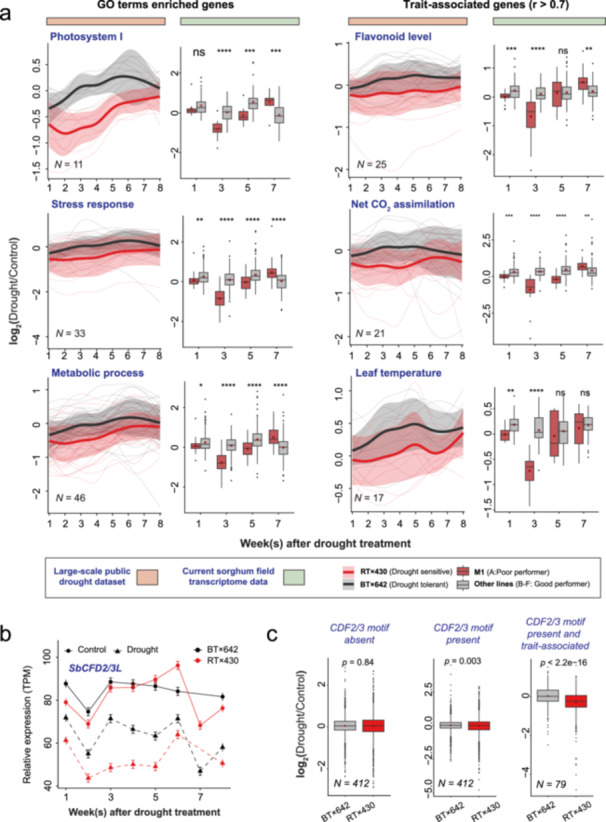
Transcriptomic response of key ‘Darkmagenta’ genes in two datasets. (a) Time‐course transcriptomic profiles of drought responses, shown as log_2_(Drought/Control), derived from a large‐scale public sorghum drought dataset (Varoquaux et al. 2019) and the current study. Genes were grouped based on GO term enrichment (left panels) or trait association (*r* > 0.7, right panels). For the public dataset, temporal trends were compared between a drought‐tolerant (BT × 642) and sensitive genotype (RT × 430) using smoothed spline curves with mean values (bold lines) and standard error (shaded area) displayed. For the current dataset, gene expression distributions at each time point are shown as boxplots, with statistical comparison between accession ‘A’ and the other accessions using the Wilcoxon rank‐sum test (*p* < 0.05). (b) Relative expression (TPM) of *SbCDF2/3L* in the public dataset over the 8 weeks of the post‐flowering drought experiment between two genotypes under control and drought conditions. (c) Comparison of log_2_(Drought/Control) expression changes between the drought‐tolerant genotype BT × 642 and the drought‐sensitive genotype RT × 430 across three gene categories: genes lacking predicted CDF3 binding motifs, genes containing predicted CDF2/3L binding motifs, and genes that both contain CDF2/3L binding motifs and show significant correlations with photosynthetic traits using a Wilcoxon rank‐sum test (*p* < 0.05). [Color figure can be viewed at wileyonlinelibrary.com]

We next aimed to determine the robustness of the observed relationship between SbCDF2/3L, its suite of putative target genes, and performance under drought conditions in sorghum. To do so, we analysed a publicly available sorghum drought time‐course RNA‐seq dataset (Varoquaux et al. 2019), quantifying relative gene expression (TPM) between a post‐flowering drought‐tolerant (BT × 642) and a drought‐sensitive (RT × 430) genotype, focusing on core genes identified within the ‘Darkmagenta’ module in our field‐derived data. Initially, we grouped the GO term‐enriched genes into three classes (Photosystem I, stress response and metabolic process; Figure 5a; left) and three groups of genes associated with oxidative stress levels and photosynthetic traits, including flavonoid level, net CO_2_ assimilation and leaf temperature (surface; Figure 5a; right). We then used the relationship between abundance under treatment and control conditions [log_2_(Drought/Control)] to represent the variation in the treatment response of these six groups of genes over the timespan of the experiment (7 weeks and 8 weeks in the current study and the public data, respectively). This comparative analysis demonstrated that in both experiments, the drought‐resilient accessions are more likely to harbour elevated expression levels of these traits and stress‐response‐associated transcripts under drought conditions. In contrast, both poor‐performing accessions (‘A’ and ‘RT × 430’) displayed reduced levels of transcripts in all six groups of genes with high GS or MM in the ‘Darkmagenta’ module (Figure 5a). These data suggest that poor‐performing sorghum accessions are unable to elicit a stress response and cannot maintain optimal photosynthetic efficiency under drought conditions. In addition, given the overlap between these two experiments, these genes are likely important markers of a robust drought response in sorghum.

Given the connection between these trait‐associated genes and SbCDF2/3L in the ‘Darkmagenta’ module, we next examined the expression profile of *SbCDF2/3L* in the drought‐tolerant genotype BT×642 and the drought‐sensitive genotype RT×430. Although *SbCDF2/3L* expression was down‐regulated in response to drought in both genotypes, the drought‐sensitive genotype exhibited significantly lower expression levels compared with the tolerant genotype over time (Figure 5b, *p* < 0.05). This pattern is consistent with the reduced *SbCDF2/3L* expression observed in accession ‘A’ relative to the other five genotypes (Figure 4b), supporting a critical role for *SbCDF2/3L* in drought‐responsive regulation in sorghum. We further assessed drought‐responsive expression differences in this public dataset between genes predicted to contain, versus those lacking CDF2/3 binding motifs within the ‘Darkmagenta’ module (Figure 5c). Genes harbouring putative CDF2/3 binding motifs displayed significantly higher log_2_(Drought/Control) ratios in the drought‐tolerant BTx642 genotype compared with the sensitive genotype (*p* = 0.003), whereas no significant difference for genes without CDF2/3 binding motifs in their 2‐kb promoter regions (*p* = 0.84). Interestingly, this drought response pattern was further amplified when restricting the analysis to genes that both contain predicted CDF2/3 binding motifs and are correlated with LI‐6800–derived photosynthetic traits (*p* < 2.2e−16). This pattern is consistent with the transcriptomic response in our field data (Figure 4c,d). Collectively, these cross‐experiment analyses using two independent datasets corroborate *SbCDF2/3L's* role as a potential positive regulator of photosynthetic and stress response genes in sorghum.

## 4 Discussion

Drought stress typically induces complex phenotypic and molecular responses that often lead to reduced biomass, seed set and photosynthesis, thereby adversely affecting the overall productivity of agricultural crops (Lesk et al. 2022). Sorghum is generally well adapted to hot and dry environments, although there is variation in how different sorghum accessions perform, variation that can be leveraged to shed light on the exact regulatory mechanisms by which sorghum achieves maximal growth under these conditions. Given the complex nature of plant responses to water deprivation, a variety of stress‐responsive genes have been identified in sorghum and other plant species (Johnson et al. 2015; Pardo and VanBuren 2021; Pardo et al. 2023; Marks et al. 2024). However, induction of a transcriptional regulator does not imply that its downstream response is beneficial to plant growth and yield. Thus, we still lack an understanding of which of these regulatory mechanisms and their targets contribute to improved agricultural traits in a field setting and therefore could serve as breeding markers or targets for manipulation.

**Figure 6:**
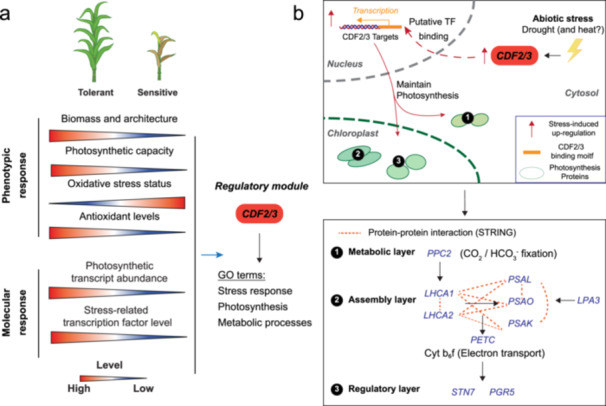
Conceptual model illustrating drought‐responsive regulatory networks in sorghum. (a) Integration of multi‐omic datasets to identify drought‐responsive co‐expression networks and a potential regulatory module connecting drought response to photosynthesis. Differences between drought‐tolerant and drought‐sensitive genotypes are reflected at both phenotypic (biomass, plant architecture, photosynthetic capacity, antioxidant levels and oxidative stress status) and transcriptomic levels, which lead to the identification of the candidate regulator CDF2/3, which is co‐expressed alongside a suite of genes involved in stress response, photosynthesis and metabolic processes. (b) Proposed regulatory model in which CDF2/3 binds and regulates stress‐responsive genes within the identified module, including a subset of nuclear‐encoded photosynthesis‐related genes correlated with LI‐6800‐derived traits (top). Many of these genes have been shown to physically interact, a subset of which is shown (bottom) due to their roles in carbon fixation (CO_2_/HCO_3_^−^ metabolism), photosynthetic complex assembly, electron transport and the chloroplast‐level regulation of photosynthetic performance under stress. Protein–protein interactions (PPIs) inferred from the STRING database are indicated by orange dashed lines (bottom). [Color figure can be viewed at wileyonlinelibrary.com]

We used a representative subset of the genetic and phenotypic diversity found within sorghum to identify molecular mechanisms that might explain the reported resilience to hot and dry environments in this species. Our panel of six distantly related sorghum accessions, grown under hot and arid field conditions, revealed distinct physiological and molecular responses to hot and arid conditions, with some accessions exhibiting minimal reductions in biomass and photosynthetic efficiency, whereas others (e.g., ‘A’) performed quite poorly. Leaf‐level physiological traits tracked closely with oxidative stress status across the six accessions, allowing us to make inferences as to which molecular mechanisms were contributing to phenotypic success (Figure 6a). Interestingly, pairwise comparisons of transcriptomic profiles between accessions and conditions revealed little overlap, suggesting highly variable responses to the environment. Thus, we utilised a comparative systems‐level approach to identify cohorts of transcripts whose expression patterns were most closely linked to molecular and physiological responses to drought stress (Figure 4, Figure 6a).

One co‐expression module in particular exhibited variation due to both treatment and genotype, signified by a contrasting response between poor and strong performers (Figure 3b). This module was enriched for genes involved in photosynthetic processes and abiotic stress responses, consistent with the central role of photosynthetic status as a key physiological indicator of drought stress (Pinheiro and Chaves 2011; Zargar et al. 2017; Zahra et al. 2023). At the regulatory level, we identified one stress‐induced TF in particular that was positively associated with photosynthetic traits and plant performance (*SbCDF2/3L;* Figures 3 and 4). While originally annotated as SbDof8 (Kushwaha et al. 2011), we found that this TF clusters with, and harbours similar Dof motifs as, *Arabidopsis* and rice CDF2/3 orthologs. Indeed, promoter enrichment and correlation between SbCDF2/3L and target genes across two independent experiments, as well as the lack of other CDF or Dof homologues in the module, suggest that SbCDF2/3L is likely a key regulator at the junction between photosynthesis and drought stress responses in sorghum (Figure 6a). This assumption is further supported by extensive evidence from previous studies demonstrating coordinated regulation between drought‐responsive TFs and photosynthesis‐associated target genes under drought conditions (Zhao et al. 2017; Muhammad et al. 2020; Bai et al. 2025). Further functional validation will, of course, be critical in confirming this model. Of note, a number of Dofs have been found to modulate responses to the environment across the land plant lineage. However, the circadian nature of the *Arabidopsis* CDFs has only been demonstrated for a few grass Dof TFs, such as RDD1 (Iwamoto et al. 2009; Y. Zhang et al. 2023), and whether this is a common feature of CDF homologues such as SbCDF2/3L and is a crucial aspect of their ability to integrate plant growth under stress conditions remains to be determined.

By closely examining photosynthesis‐related responses to water limitation in two independent experiments, we delineated the transcriptomic patterns underlying differential drought responses in drought‐tolerant and sensitive genotypes (Figure 5a). We identified a subset of genes that were both strongly correlated with LI‐6800‐derived photosynthetic traits and significantly enriched for photosynthesis‐related GO terms (Figures 4e and 5a). Notably, these genes span multiple layers of the photosynthetic process, indicating that drought‐induced reductions in photosynthesis are not confined to a single pathway but involve coordinated perturbations across the photosynthetic machinery. Many of these photosynthetic trait‐correlated genes are known to be responsive to abiotic stress (Figure 6b). For instance, LHCA proteins involved in capturing and transferring solar energy have been shown to influence drought tolerance in barley, fava bean, cotton and other species (Mehari et al. 2021; Cheng et al. 2025; Huang et al. 2025). Furthermore, *Psao*, *Psak* and *Psal* encode PSI subunits that cooperate with LHCA proteins to stabilise PSI machinery and sustain electron transport under drought stress (Seok et al. 2014; Dudhate et al. 2018). In drought‐tolerant plants, PETC generally exhibits a delayed and attenuated downregulation, likely due to improved ROS homoeostasis and sustained D1 protein turnover (Silva et al. 2024). At the regulatory level, *STN7* links photosynthetic complex formation and leaf senescence in *Arabidopsis* (Lee et al. 2025), and PGR5 is a key regulator of PSI cyclic electron transport and supports photosystem protection under drought stress (Guangxin et al. 2025). Thus, these genes represent a concerted effort to combat drought stress, one that appears to be regulated largely by a plant‐specific TF, SbCDF2/3L and one that could be promising for further investigation in the context of drought‐induced photosynthesis improvement in sorghum. We therefore propose a conceptual model in which modulating a key stress response TF, SbCDF2/3L, specifically under drought conditions, may have positive effects on both drought tolerance and photosynthetic capacity (Figure 5b). Dissecting and potentially enhancing this transcriptional regulation in the future offers a promising avenue to sustain photosynthetic performance under WL conditions, thereby providing a translational framework for improving drought resilience, biomass accumulation and yield stability in sorghum production.

## Supporting information


**Figure S1:** Physiological response of drought tolerance in sorghum accessions. (a) Microbiome features, represented by 515 classified bacterial taxa and 46 fungal taxa, showing intra‐accession variation based on principal component analysis (PCA) at the terminal sampling stage. (b) Boxplots show changes of biomass and root phenotypes (Drought/Control) among six accessions using Kruskal test. (c) Bar plots showing photosynthetic efficiency and capacity under control conditions, measured as Fv′/Fm′ and net CO_2_ assimilation, respectively, at each time point for drought‐treated plants (*N* = 10 per accession). Pairwise Wilcoxon tests were used to compare each accession relative to “A” (*p* < 0.05). **Figure S2:** Transcriptome response of field‐derived sorghum samples. (a) A hierarchical clustering (Pearson correlation) of 34,130 transcripts normalized by DESeq. 2 was used to group transcriptomic features among the 96 samples taken over the course of the experiment (Weeks 1, 3, 5, 7; drought vs control). The scale indicates the Pearson correlation coefficient (PCC) score. (b) PCA plot using top 10% most variable genes. (c, d) Comparisons of the down‐regulated (left) and up‐regulated (right) genes among samples per genotype across each later weeks to the first week of sampling. Numbers of DEGs were displayed using the upset plots. The top bar charts denote the shared (or unique) DEGs corresponding to the inclusion of samples below, and bottom‐left bars denote the total numbers of DEGs derived from each comparison. **Figure S3:** Stress response profiling of four antioxidant products. Boxplots show changes of antioxidant metabolites and enzymes (Drought/Control) over four weeks among six accessions using Kruskal test. **Figure S4:** Phylogenetic tree of Dof gene family across species. Maximum‐likelihood (ML) tree was constructed using RAxML with Dof domain genes identified in sorghum, rice, and *Arabidopsis*. Dof nomenclatural assignment comes from prior publications (see Results). **Figure S5:** Expression profile of selected Dof genes in sorghum. Relative expression (TPM) of four CDF‐like Dof genes in sorghum across six accessions over four timepoints under control and drought conditions.

**Table S1:** GO enrichment of DEGs from later (week 7) vs. initial stage (week 1) under both watering conditions. **Table S2:** WGCNA result summary. **Table S3:** Pearson correlation of WGCNA‐module to Licore‐6800 data. **Table S4:** Pearson correlation of WGCNA‐module to oxidative enzyme activity. **Table S5:** Selection of hub TFs of darkmagenta module based on AME. **Table S6:** Darkmagenta module statistics.

## Data Availability

Transcriptome sequencing reads are available for download from the National Center for Biotechnology Information (NCBI) BioProject database under the accession number: PRJNA1119650. Computational pipelines used for data analysis were deposited under the GitHub page (https://github.com/Leon-Yu0320/Sorghum_Range28). The R markdown files (R code) for data visualisation in figures were deposited in the Rpubs workspace (https://rpubs.com/LeonYu/Sorghum-comparative-transcriptomics).
