# Supplementary material for: Integrated Multi‐Omic Analyses Uncover a Regulatory Link Between Photosynthesis and Drought Tolerance in Field‐Grown Sorghum
Source: Plant Cell Environ. 2026 Jun 7;49(9):6748–64. doi: 10.1111/pce.70649 (PMC13436492; doi:10.1111/pce.70649)
Supplement: Supplementary file 1 [file PCE-49-6748-s002.pdf]

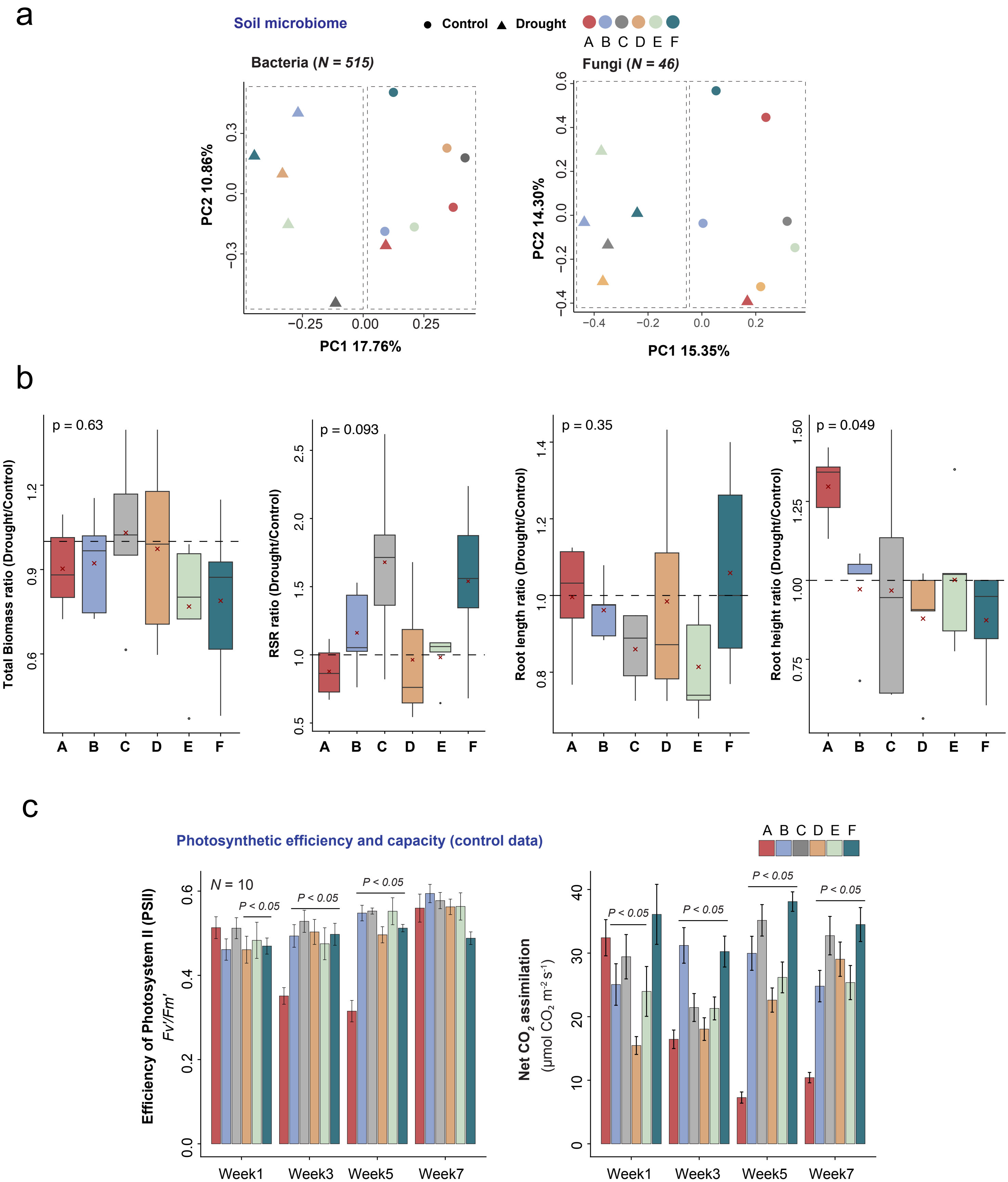

a
Soil microbiome
Control
Drought
A B C D E F
Bacteria (N = 515)
Fungi (N = 46)
PC2 10.86%
PC1 17.76%
PC2 14.30%
PC1 15.35%
b
p = 0.63
p = 0.093
p = 0.35
p = 0.049
Total Biomass ratio (Drought/Control)
RSR ratio (Drought/Control)
Root length ratio (Drought/Control)
Root height ratio (Drought/Control)
c
Photosynthetic efficiency and capacity (control data)
N = 10
P < 0.05
Efficiency of Photosystem II (PSII)
Fv'/Fm'
Net CO2 assimilation
(μmol CO2 m-2 s-1)
Week1
Week3
Week5
Week7

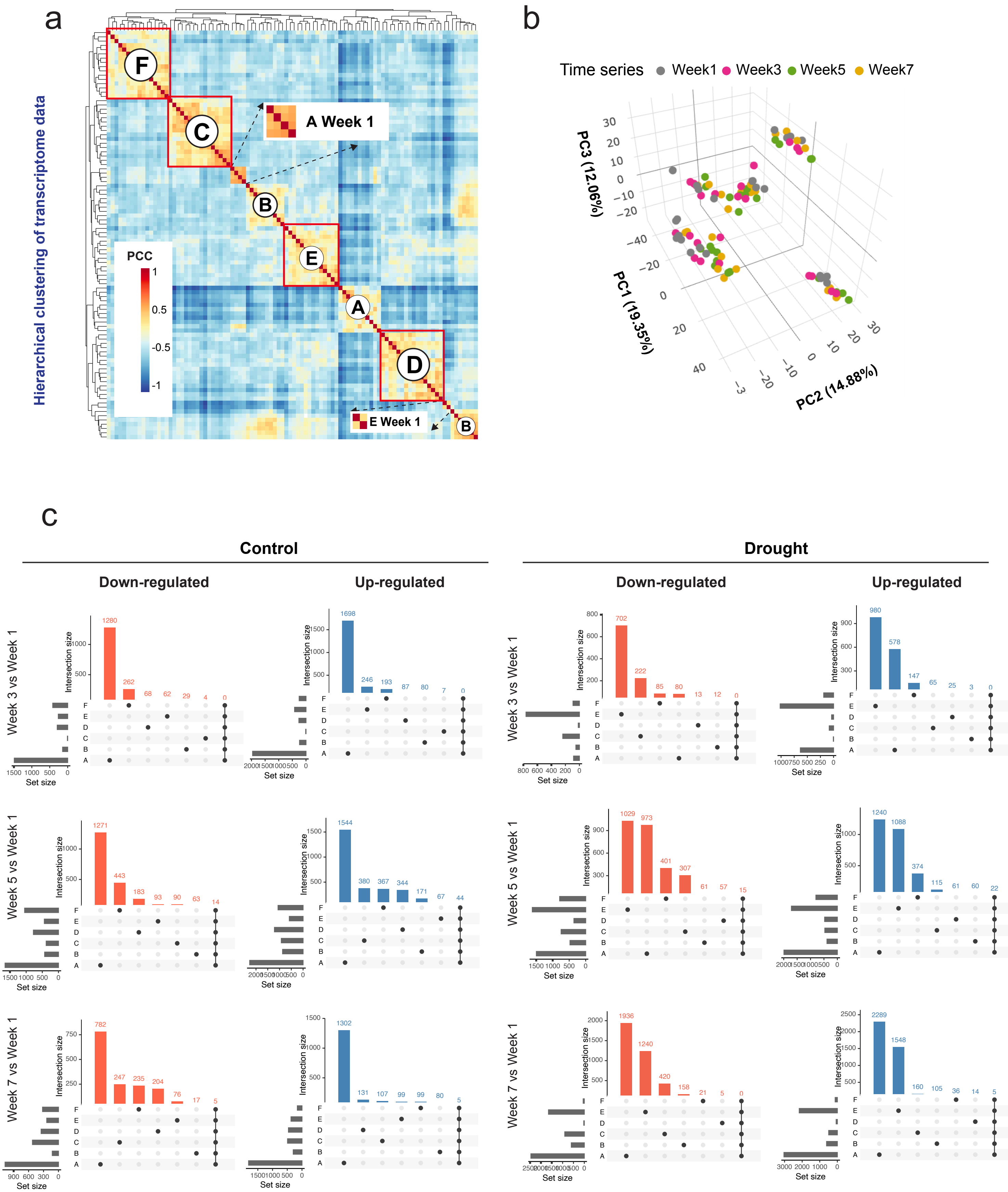

a
Hierarchical clustering of transcriptome data
PCC
1
0.5
-0.5
-1
F
C
A Week 1
B
E
A
D
E Week 1
B
b
Time series
Week1
Week3
Week5
Week7
PC3 (12.06%)
PC1 (19.35%)
PC2 (14.88%)
c
Control
Drought
Down-regulated
Up-regulated
Week 3 vs Week 1
Week 5 vs Week 1
Week 7 vs Week 1
Intersection size
Set size
1280 262 68 62 29 4 0
1698 246 193 87 80 7 0
702 222 85 80 13 12 0
980 578 147 65 25 3 0
1271 443 183 93 90 63 14
1544 380 367 344 171 67 44
1029 973 401 307 61 57 15
1240 1088 374 115 61 60 22
782 247 235 204 76 17 5
1302 131 107 99 99 80 5
1936 1240 420 158 21 5 0
2289 1548 160 105 36 14 5

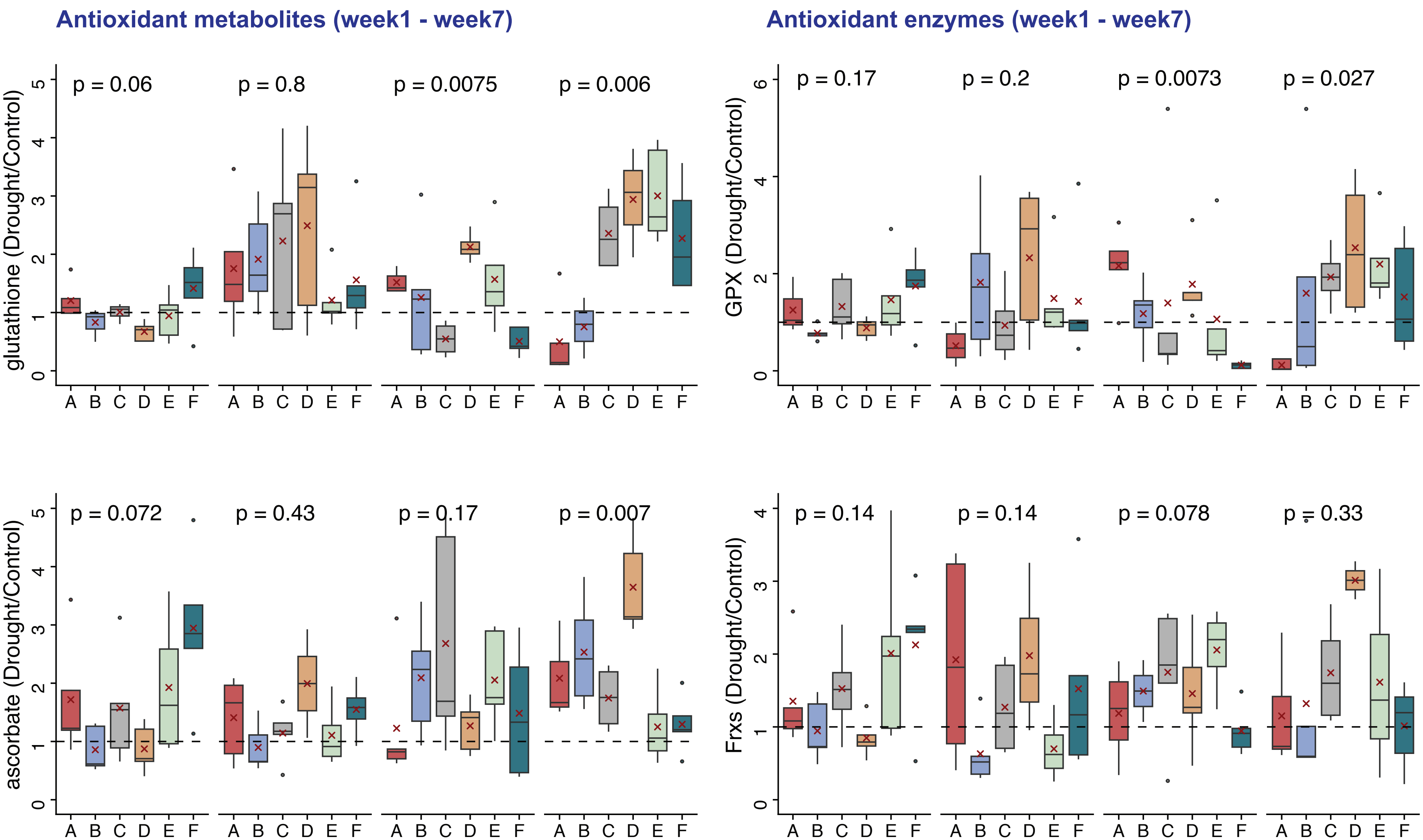
Antioxidant metabolites (week1 - week7)
Antioxidant enzymes (week1 - week7)
glutathione (Drought/Control)
p = 0.06
p = 0.8
p = 0.0075
p = 0.006
GPX (Drought/Control)
p = 0.17
p = 0.2
p = 0.0073
p = 0.027
ascorbate (Drought/Control)
p = 0.072
p = 0.43
p = 0.17
p = 0.007
Frxs (Drought/Control)
p = 0.14
p = 0.14
p = 0.078
p = 0.33
A B C D E F

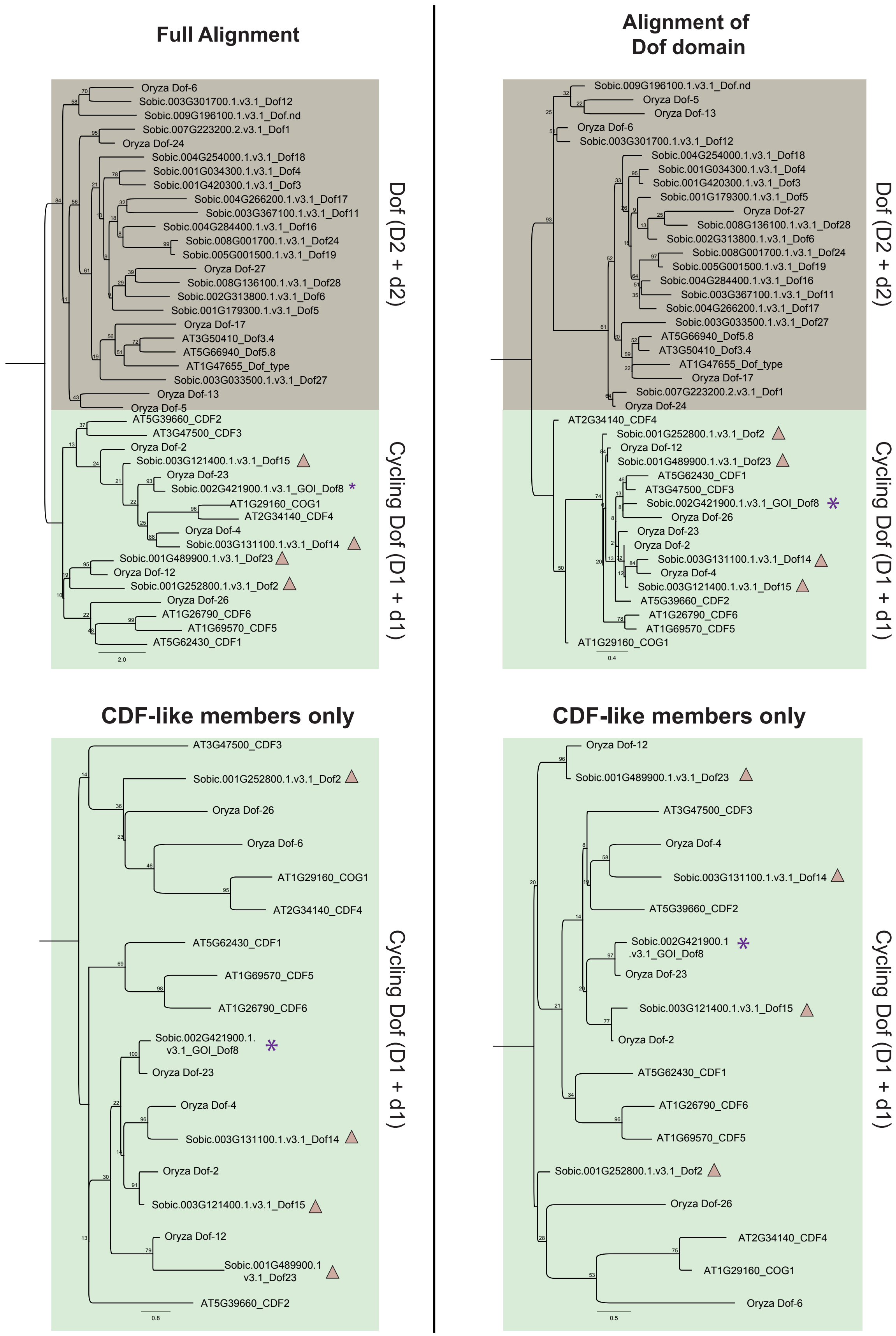

Full Alignment
Alignment of Dof domain
Oryza Dof-6
Sobic.003G301700.1.v3.1_Dof12
Sobic.009G196100.1.v3.1_Dof.nd
Sobic.007G223200.2.v3.1_Dof1
Oryza Dof-24
Sobic.004G254000.1.v3.1_Dof18
Sobic.001G034300.1.v3.1_Dof4
Sobic.001G420300.1.v3.1_Dof3
Sobic.004G266200.1.v3.1_Dof17
Sobic.003G367100.1.v3.1_Dof11
Sobic.004G284400.1.v3.1_Dof16
Sobic.008G001700.1.v3.1_Dof24
Sobic.005G001500.1.v3.1_Dof19
Oryza Dof-27
Sobic.008G136100.1.v3.1_Dof28
Sobic.002G313800.1.v3.1_Dof6
Sobic.001G179300.1.v3.1_Dof5
Oryza Dof-17
AT3G50410_Dof3.4
AT5G66940_Dof5.8
AT1G47655_Dof_type
Sobic.003G033500.1.v3.1_Dof27
Oryza Dof-13
Oryza Dof-5
AT5G39660_CDF2
AT3G47500_CDF3
Oryza Dof-2
Sobic.003G121400.1.v3.1_Dof15
Oryza Dof-23
Sobic.002G421900.1.v3.1_GOI_Dof8 *
AT1G29160_COG1
AT2G34140_CDF4
Oryza Dof-4
Sobic.003G131100.1.v3.1_Dof14
Sobic.001G489900.1.v3.1_Dof23
Oryza Dof-12
Sobic.001G252800.1.v3.1_Dof2
Oryza Dof-26
AT1G26790_CDF6
AT1G69570_CDF5
AT5G62430_CDF1
2.0
Dof (D2 + d2)
Cycling Dof (D1 + d1)
0.4
CDF-like members only
Sobic.002G421900.1.v3.1_GOI_Dof8
0.8
Sobic.001G489900.1 v3.1_Dof23
0.5

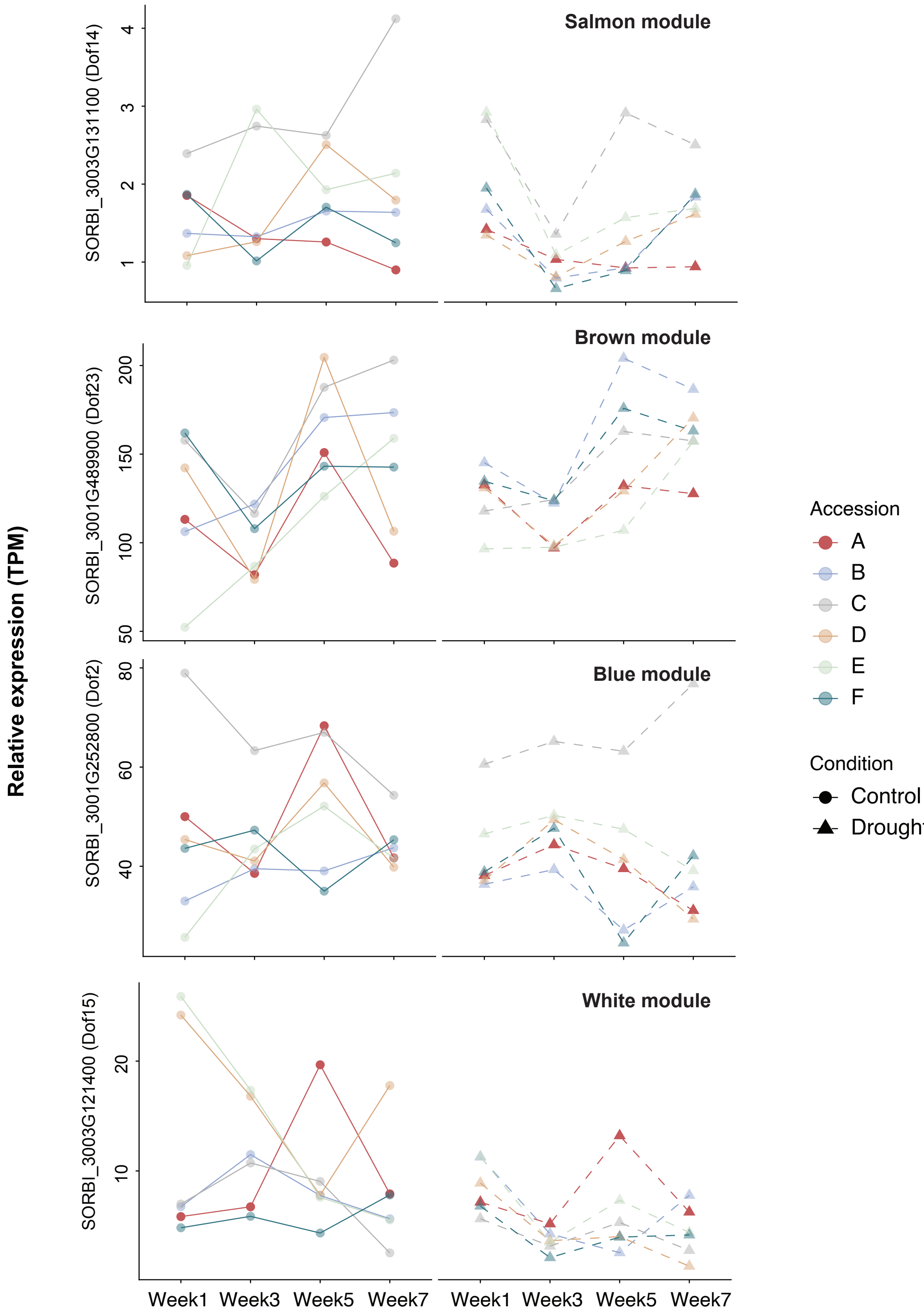

Relative expression (TPM)
Salmon module
SORBI_3003G131100 (Dof14)
Brown module
SORBI_3001G489900 (Dof23)
Blue module
SORBI_3001G252800 (Dof2)
White module
SORBI_3003G121400 (Dof15)
Week1
Week3
Week5
Week7
Accession
A
B
C
D
E
F
Condition
Control
Drought
